# A Selection of Starting Points for Iterative Position Estimation Algorithms Using Feedforward Neural Networks

**DOI:** 10.3390/s24020332

**Published:** 2024-01-05

**Authors:** Jaroslaw Sadowski, Jacek Stefanski

**Affiliations:** Faculty of Electronics, Telecommunications and Informatics, Gdansk University of Technology, 80-233 Gdansk, Poland; jstef@eti.pg.edu.pl

**Keywords:** radio localization, position estimation, iterative algorithms, feedforward neural network

## Abstract

This article proposes the use of a feedforward neural network (FNN) to select the starting point for the first iteration in well-known iterative location estimation algorithms, with the research objective of finding the minimum size of a neural network that allows iterative position estimation algorithms to converge in an example positioning network. The selected algorithms for iterative position estimation, the structure of the neural network and how the FNN is used in 2D and 3D position estimation process are presented. The most important results of the work are the parameters of various FNN network structures that resulted in a 100% probability of convergence of iterative position estimation algorithms in the exemplary TDoA positioning network, as well as the average and maximum number of iterations, which can give a general idea about the effectiveness of using neural networks to support the position estimation process. In all simulated scenarios, simple networks with a single hidden layer containing a dozen non-linear neurons turned out to be sufficient to solve the convergence problem.

## 1. Introduction

Positioning services in radio networks allow for estimating the position of a mobile terminal (object) using radio waves. Most radio positioning systems are based on two main properties of radio wave propagation. The first is propagation along straight lines in a homogeneous environment, which is the operating principle of angular positioning systems, while the second is the limited variability of radio wave propagation speed in most environments. However, no matter what the basis of a given localization system is, the implementation of measurements of radio signal parameters rarely provides directly useful localization or navigation information, because users expect the data to be presented in a form that is convenient for them. Therefore, the tasks performed by positioning systems and devices can be divided into two phases. The first phase is the measurement of radio signal parameters, such as angle of arrival, time of arrival, carrier phase, Doppler shift frequency and even the channel impulse response. The choice of parameters to be measured depends on variety of factors, including system structure, frequency band, signal shape and modulation, antennas, the computational power of the equipment used and the expected performance of the localization system. However, a discussion of measurement methods is beyond the scope of this article.

The second phase is the conversion of the measurement results into useful positional or navigational data. It may include the calculation of the coordinates of the mobile terminal in a global Cartesian coordinate system, one of many geographic and geodetic coordinate systems or some local reference system, but also the calculation of the speed, course, bearing and path to a specific waypoint. In most systems and measurement methods, the relationship between measurement results and mobile terminal coordinates is non-linear, and, in some solutions, also ambiguous. For this reason, various methods and algorithms for calculating positions have been developed, some of which are based on general algorithms for solving systems of non-linear equations or finding zeros of non-linear continuous functions [1,2], while others have been developed strictly for use in positioning systems [3,4,5]. In general, these algorithms can be divided into two groups: closed-form algorithms, which allow the position of the mobile terminal to be calculated by performing a specific mathematic operation (or sequence of operations) once, and iterative algorithms, which involve repeated calculations, in many cases using an approximation of non-linear equations with linear functions.

The algorithms from the first group always return results after a finite set of operations, and these results are final estimates of the position and/or navigation data, which may be equal to true data in the case of error-free measurements or estimates with errors in the case of imperfect measurements. On the other hand, iterative algorithms return approximate solutions that are generally improved with each iteration; but, in theory, as long as the number of iterations is finite, the results of iterative algorithms are never an exact solution to the positioning problem, no matter how accurate the measurements are. Of course, in practical implementations, the number of iterations is finite and rarely exceeds a few tens of calculation repetitions. Iterations are usually terminated when the error of the positioning equations falls below a certain threshold or the maximum number of repetitions is reached. Even with only a few iterations, the total number of mathematical operations performed by iterative algorithms easily exceeds the computational complexity of closed-form algorithms and it is often cited as one of major drawbacks of iterative position calculation. The second major drawback is the convergence problem. Since all iterative algorithms increase the quality of the estimated parameters (e.g., coordinates) at every iteration, there must be some initial set of parameters for the first iteration of calculation. The selection of these initial values, also referred to as the starting point, influences the number of iterations required to reach a certain threshold of calculation error, with the general rule being that the closer the starting point is to the true position of the estimated object, the fewer iterations are needed. Unfortunately, it is not guaranteed that all iterative algorithms will always converge to the correct solution, typically understood as the minimum error of the positioning equations expressed by the global minimum of the error function. Convergence to some local minima of error function is also possible, leading to incorrect position estimation results, and in certain conditions algorithms may even diverge to infinity. It is commonly known that the closer the starting point is to the real coordinates of the mobile terminal, the greater the chance of a correct convergence of the position estimation results to the actual values, but it is difficult to find a universal method for selecting the starting point. Various methods can be used in practice, with a fixed starting point selected as an average value of coordinates of reference nodes being the most obvious one. In the case of mobile terminal tracking, a good starting point for iterative position estimation is usually a previously estimated position obtained during earlier measurements, as the dynamics of motion are usually limited. The problem increases when the position of the mobile terminal is estimated once, without any support from earlier measurements or other sources of information. One can imagine selecting several starting points for iterative position estimation and comparing results from several instances of iterative algorithms running in parallel, or calculating the initial coordinates using some closed-form algorithm. In such a case, the question arises: why bother with iterative algorithms?

There are examples where iterative position calculation significantly outperforms closed-form algorithms. An interesting discussion on closed-form algorithms and their drawbacks can be found in [6], where the author lists the following problems with some of the algorithms presented in the literature: calculations based on squared equations lead to the minimization of positional equations’ errors raised to the fourth power, which yields results different than in those in the case of the least squares method; auxiliary variables being treated as independent variables, while they strictly depend on already existing unknowns; and poor performance in the case of correlated errors. Of course, not all the problems listed above are applicable to all examples of closed-form algorithms, but one assumption is common to most of them: the Gaussian distribution of measurement errors. In the case of non-Gaussian error distribution, and especially when the measurement error distribution is asymmetric, it is much easier to account for this in iterative algorithm than in closed-form algorithms. For example, in [7], an iterative position estimation algorithm for a distance-based positioning system was proposed that differentiates the weights of the measurement results in each iteration and takes into account the uneven distribution of round-trip time (RTT) errors in the case of non-line of sight (NLOS) propagation. However, the advantages of iterative calculations are not limited to these. There are also cases where the structure and coefficients of the positioning equations depends on the position of the mobile terminal, which is to be estimated. An example of such a case may be a non-linear relation between distance and radio signal propagation time in the case of propagation through a boundary between environments with different characteristics. This may include a different speed of ground waves propagating over land and over salty sea in the Loran system, a non-linear relation between the carrier phase and distance in the near field of a transmitting antenna, or the non-linear relation between the length of the propagation path around the curvature of Earth’s surface and the Cartesian distance between terminals. Such non-linearities are easily handled in iterative algorithms, but not in closed-form ones. Another example would be a 2D positioning system in which reference nodes are not located on the same plane as the mobile terminals. In the case of distance measurements, e.g., using time of arrival (TOA) or RTT methods, the results of measurements taken in a 3D environment can be easily converted to distances in a 2D scenario in one step, using geometric relationships. However, in the case of distance-difference measurements, e.g., by the time difference of arrival (TDoA) method, the conversion from 3D measurements to 2D data for position estimation depends on the position of the mobile terminal. Therefore, iterative computation is almost inevitable. A final argument in favour of using iterative position estimation algorithms is that data from different measurement methods, for example distance and angle-based, can be easily integrated.

Since there are systems and solutions where iterative position calculation is not only more convenient, but even unavoidable, the focus should be on eliminating, or at least reducing the drawbacks of, iterative algorithms. This article will consider the problem of the starting point for the first iteration and its impact on the convergence of selected iterative position calculation algorithms. Some methods for selecting the starting point have already been mentioned above, but the authors would like to use feedforward neural networks (FNN) as a coarse position estimation tool that can be used as a starting point for fine calculations using iterative algorithms. In recent years, many examples of the use of neural networks in positioning have been proposed in the literature, some of which will be briefly presented in Section 2. In these publications, a lot of effort has been put into ensuring that neural networks are able to return the most accurate position estimates, so the neural networks replace all the data processing typically performed by position calculation algorithms. However, in this publication, the feedforward neural network is only responsible for the approximate estimation of the mobile terminal position and the aim is to find the simplest structure of FNN that allows us to achieve a convergence of final position estimations using iterative algorithms over the entire area of system operation.

This paper is organized as follows: Section 2 contains the state of the art in using neural networks in positioning. The system model, used to test the convergence of iterative position estimation algorithms and the structure of the neural network, are described in Section 3. The next section contains simulation results with comments, while conclusions and ideas for further research are presented in Section 5.

## 2. Related Works

Radio positioning systems and networks can be divided into two groups. The first group includes positioning methods and techniques that do not require the determination of strict geometrical relationships between nodes, such as distances or bearing angles. Systems and solutions based on channel state information, neighborhood detection and fingerprinting (“range-free positioning”) are gaining much attention [8,9,10,11]. In many cases, there are no direct relationships or general equations that can be used to relate the results of radio signal parameters measurements to the coordinates of the mobile terminal in this group of networks. Therefore, the use of neural networks for position estimation, including deep-learning solutions, seems to be a perfect solution. For example, in [12], a feedforward neural network was used to estimate the position in a network based on Wi-Fi access points with the fingerprinting principle. The authors of [12] focused on the network training algorithm, which, together with the training dataset, has a great impact on the performance of the neural network, and they proved that Wi-Fi-based fingerprinting can provide 1 to 4 m positioning accuracy in indoor environments. Similar accuracy can also be found in [13].

More comprehensive research can be found in [14], where a neural network was used to estimate the position in a fingerprinting-based system that used measurements from two Wi-Fi bands. Different scenarios were tested in [14], with different neural network structures and network training datasets, with the conclusion that the quality of position estimation using data from two Wi-Fi bands outperforms the single band case. In [15], the position of the mobile terminal was estimated using the downlink signals of both Wi-Fi and LTE. The results of the received signal strength (RSS) measurements were processed by several neural networks: the first one was used to determine the indoor/outdoor scenario, the second was used to detect the floor number in the indoor case, and finally separate networks were used to estimate the position on each floor and in an outdoor environment. The article [15] was based on real field measurements, but in [16], only simulated values of RSS data were used for both training and evaluating two neural network models for position estimation in a sensor network. Therefore, the results presented in [16] may be affected by the use of an inaccurate path loss model that differs from actual signal attenuation in a real environment. In comparison, [17] should not have such flaws, since it was based on measurements of signal levels from Xbee modems in a real indoor environment. The authors did not specify in which ISM band the modems operated, but four neural network structures were examined: linear regression, random forest, K-nearest neighbor and decision tree, and the article includes a comparison of their position estimation accuracy. Xbee modems and RSS measurement data were also used in [18], where very small neural networks with three, four or five non-linear neurons in single hidden layer were used, but in this publication both the training and the test dataset were very limited, too small to generalize conclusions.

In addition to papers describing the results of real field measurements, publications based solely on simulations can also be found. For example, in [19], the received signal power for the 5G mmWave band (28 GHz) in an outdoor environment was estimated in simulations using raytracing, and a multilayer perceptron (MLP) neural network was used to estimate the position of the mobile terminal based on RSS estimates. In this case, the usability of the presented results mainly depends on the accuracy of the environment modelling in the simulations.

In some of the abovementioned positioning systems with artificial intelligence that used fingerprinting principle, the results of position estimation may differ from the reference points that were used to collect the “fingerprints” because the neural network can perform some interpolation of the data. However, in [20], a neural network was used to classify measurement data into one of twenty-six possible locations in an indoor test environment. This solution used Bluetooth Low Energy (BLE) tags to transmit radio packets, which were received by five stations that performed an initial analysis of the RSS results, calculating the average value, maximum/minimum, root-mean-square, standard deviation, skewness and kurtosis. All these data were used as input data for a feedforward neural network with two hidden layers, and the probability of correct classification was evaluated as position estimation efficiency. A similar approach can be found in [21], where RSS measurements from the EnOcean radio network (922 MHz) were used to detect in which room the mobile terminal was placed.

Many more examples of using machine learning in indoor positioning using the Wi-Fi radio interface can be found in very extensive review articles [22,23]. It is somehow interesting that majority of the solutions mentioned in both these publications rely on RSS and channel state information (CSI) estimation, with no angle- or time-based solutions. However, some examples of RSS-based ML positioning experiments allowed for reducing error in indoor environments below one meter, which is very promising. In general, most of the ML-based positioning proposed in the abovementioned publications use a fingerprinting method based on existing Wi-Fi, and sometimes LTE signals, which is in accordance with a survey presented in [24], but definitely a wider range of applications of neural networks in position estimation is possible. A deep look into the problem of using deep learning in fingerprinting-based position estimation networks can be found in [25], in which various solutions using different sources of measurement data (e.g., Wi-Fi, BLE and cellular networks radio fingerprints, magnetic fields and image recognition) and various neural networks’ algorithms are discussed. Some publicly available datasets with fingerprints for indoor positioning testing and development are also presented in [25].

In the second group of positioning systems, the position of the mobile terminal is estimated using measurements of the geometrical parameters of the radio network. For example, in [26], machine learning was used to estimate the distance between a user terminal and a Wi-Fi access point using round-trip-time principle in fine timing measurement (FTM) protocol. Compared to a maximum-likelihood estimator (MLE), the neural network-based RTT estimation gave significantly lower error values, but the article lacks a discussion of what made the ML-based distance estimation so significantly better.

An interesting approach to using a neural network to integrate various data without explicitly known relationships between them is presented in [27]. In addition to signal power measurements in the 868 MHz band, a link quality indicator (LQI) and temperature and humidity measurements were used to estimate distances between nodes using a neural network for position estimation. However, it is difficult to imagine that temperature and humidity could affect the quality of distance estimation, but this article does not provide any comparison of the results with and without taking these measurements into account.

Another position estimation method that takes advantage of artificial neural networks is presented in [28]. In this solution, a single 5G base station was equipped with two antenna arrays with 64 antenna elements each, which performed digital beamforming to create 48 beams. The mobile terminal measured beam reference signal received power (BRSRP) data, which were processed by the neural network and random forest algorithm together with direction of departure (DoD) data from the beamformer to estimate a terminal position in an urban area, with additional data from a LOS-NLOS detector. A similar setup with one base station equipped with an antenna array is also considered in [29], but in this publication, a channel frequency response matrix was estimated as position-related data to be processed by the neural network.

Unlike other examples of positioning networks based on Bluetooth Low Energy and signal power measurements, in which a neural network was used to estimate the final coordinates of the mobile terminal, in [30], a two-layer network with 64 neurons in each hidden layer was used to estimate the distance from the smartphone to the BLE beacon. The final position estimate was calculated using the intersection points of the circles defined by the positions of the BLE beacon and the estimated distances. Surprisingly, the authors of [30] used the Rectified Linear Unit (ReLU) activation function to solve a problem of distance estimation from RSS measurements, although it only allows for obtaining an approximation of data through a piecewise linear function.

Positioning networks based on time or time-difference are not often presented in the literature in conjunction with machine learning and artificial neural networks. A good example of such a solution based on time difference of arrival (TDoA) measurements in the LoRa network is presented in [31], where a deep neural network allowed for a fourfold reduction in position estimation error by estimating and compensating TDoA results prior to position calculation. However, although the results presented in [31] are very promising, no information is given on the structure of the neural network used to process the TDoA.

The accuracy of position estimation in time-based networks decreases significantly when radio signals propagate through NLOS channels. Blocking the direct propagation path causes bias in measurement errors and a systematic shift in position estimates; that is why in [32], the support vector machine (SVM) machine-learning algorithm was used to detect LOS/NLOS conditions and change the behavior of the position calculation algorithm accordingly.

In order to facilitate the comparison of various publications describing the use of neural networks in localization, Table 1 presents a short summary of the most important information about the solutions presented in them.

Summarising the numerous examples of the use of neural networks in position estimation presented above, and many more listed, e.g., in [23,33], it should be noted that no article was found where the concept of using the result of a neural network to indicate the starting point for the first iteration in iterative position estimation algorithms is presented. Therefore, the idea presented in Section 3 and the results of computer simulations from Section 4 are novel.

## 3. System Model

It was assumed that the aim of the research was to find the simplest structure of an artificial neural network that can provide a coarse estimate of a mobile terminal position. This coarse estimate can be used as a starting point for the first iteration in iterative position estimation algorithms and should allow us to achieve convergence at every point in the defined operating area of the system.

As there are many methods for position estimation and many possible positioning network structures, which may affect the convergence of iterative calculation algorithms, further assumptions were needed.

### 3.1. Position Estimation Method

From the many different position estimation methods that can be used, e.g., in cellular networks [34,35], the time difference of arrival (TDoA) method was chosen for investigation. This is a time-based method in which the measurement results provide information about the difference in distance of the mobile object (user terminal) to two reference nodes (base stations). Thus, the measurement results in this method define hyperbolas in 2D positioning or hyperboloids in the case of 3D positioning, and the mobile terminal is located at the intersection of all hyperbolas or hyperboloids in perfect conditions or close to a group of intersection points in the presence of measurement errors. When the final position of the mobile terminal is calculated using iterative algorithms (see Section 3.3), this method is known to be more prone to an incorrect selection of the starting point for the first iteration [36], compared to positioning methods based on distance estimation (ToA, RSS) or angle estimation (AoA, AoD). However, since the TDoA positioning method has been defined as part of the 4G and 5G cellular standards, the starting point selection problem is still relevant.

### 3.2. Base Stations Geometry

A study on the selection of the starting point for iterative position estimation was carried out using the actual coordinates of five LTE base stations from one operator in Gdansk, Poland, because cellular systems already have some sort of positioning capabilities built in and they are considered sources of position data alternative to satellite positioning. The coordinates of the base stations were expressed in a local Cartesian coordinate system with the center defined by the average coordinates of all stations. The irregular distribution of base stations taken from the actual network is beneficial, as in the case of a regular grid of base stations, there could be doubt whether the obtained parameters of the minimum structure of the neural network would allow the generalization of conclusions. In fact, the symmetric deployment of base stations in simulations resulted in a much simpler network structure than in the asymmetric cases.

In two-dimensional position estimation using distance measurements, the minimum number of reference stations that allows an unambiguous indication of the mobile terminal position is three. However, in the case of distance-difference measurements, even in the simplest 2D system, all the hyperbolas defined by the positioning equations can intersect twice, causing ambiguity in some parts of area of system operation. Thus, the minimum number of base stations in 2D TDoA investigations was increased to four. For the same reasons, the 3D case requires five base stations, but most simulations were made with six stations for reasons explained in Section 4.2. The coordinates of the base stations used in all simulations are summarized in Table 2.

It is well known that the convergence problem in iterative algorithms increases in regions with higher values of the dilution of precision (DOP) parameter. In range and range-difference systems, these regions typically occur outside the area surrounded by base stations (the distance from the origin of the local coordinate system to the most distant base station was 226 m in our scenario), therefore a larger region of simulations was chosen in the form of a square of 800 m. For the 3D scenarios, the range of the *z* coordinate was from 0 to 20 m, which is the typical height of a six-story building. The convergence of the position estimation was tested by generating distance difference data corresponding to each possible position in the 2D grid with *x* and *y* ranging from −400 to +400 and a step equal to 5 m. In the case of 3D positioning, the horizontal coordinate step was set to 10 m but the vertical (*z*) was reduced to 2 m due to the smaller range of height variability. Test positions closer than 2 m from any base station were removed from the grid due to the high risk of obtaining an irreversible form of matrix at some stage of calculations in the iterative algorithms under consideration. This is not a significant reduction of system usability, as it is not common to design 4G/LTE cellular base stations in such a way that the user can approach base station antennas at a distance of less than a few meters.

It was assumed that no measurement errors were present when testing iterative position estimation algorithms. If, after a finite number of iterations, the position estimation algorithm returned coordinates within 1 m from the actual terminal position, the obtained result was evaluated as a correct convergence. If the algorithm stopped before reaching the maximum number of iterations, but the returned coordinates were further than 1 m from the correct position, a convergence to an incorrect result was counted. Finally, if the algorithm reached the defined maximum number of iterations, the results were assessed as divergent. The presence of measurement errors would make it difficult to evaluate the results, as both the starting point selection and measurement errors affect the convergence of iterative algorithms.

### 3.3. Iterative Position Calculation Algorithms

In the TDoA system that performs 2D position estimation, the actual difference in distance of the mobile terminal from a pair of base stations (reference nodes) numbered m and n can be defined as:

(1) $$d_{m,n}\left(x\right)=\sqrt{{\left(x-X_{m}\right)}^{2}+{\left(y-Y_{m}\right)}^{2}}-\sqrt{{\left(x-X_{n}\right)}^{2}+{\left(y-Y_{n}\right)}^{2}},$$

where the vector x contains the Cartesian coordinates of the tracked object:

(2) $$x={\left[x,y\right]}^{T}$$

and $X_{m},\;Y_{m},\;X_{n},\;Y_{n}$ are coordinates of the mth and nth base station, respectively. In a real system, the results of distance-difference measurements $r_{m,n}$ differ from the actual distance differences $d_{m,n}$ due to the presence of measurement errors $\varepsilon_{m,n}$. Writing both measurements and errors in vector form:

(3) $$r={\left[r_{2,1},r_{3,1},\ldots ,r_{m,n},\ldots \right]}^{T},$$

(4) $$\varepsilon ={\left[\varepsilon_{2,1},\varepsilon_{3,1},\ldots ,\varepsilon_{m,n},\ldots \right]}^{T},$$

we obtain:

(5) $$r=d\left(x\right)+\varepsilon .$$

An extension to a three-dimensional case is straightforward. The position estimation algorithm should minimize the value of a cost function:

(6) $$e\left(x\right)={\left(r-d\left(x\right)\right)}^{T}G^{-1}\left(r-d\left(x\right)\right),$$

where G is the measurement error covariance matrix. Thus, the weighted non-linear least squares solution to (6) is:

(7) $$\hat{x}=argmin_{x}e(x).$$

In iterative algorithms, the estimate of vector of coordinates $\hat{x}$ is updated iteratively, starting from an initial guess (starting point) ${\hat{x}}_{0}$. The problem of starting point selection has been mentioned, e.g., in [37], but without constructive conclusions, the authors of [37] simply used a closed-form algorithm proposed by Bancroft [38].

#### 3.3.1. Gauss-Newton

As it is clearly presented in [39], the Gauss–Newton algorithm is based on the linearization of system equations about some initial value of the vector $x_{0}$, giving the approximation:

(8) $$d\left(x\right)\approx d\left(x_{0}\right)+A\left(x_{0}\right)\left(x-x_{0}\right),$$

where $A\left(x\right)$ is Jacobian matrix. For the 2D TDoA case:

(9) $$A\left(x\right)=\left[\begin{array}{cc} \frac{x-X_{2}}{r_{2}}-\frac{x-X_{1}}{r_{1}} & \frac{y-Y_{2}}{r_{2}}-\frac{y-Y_{1}}{r_{1}}\\ \frac{x-X_{3}}{r_{3}}-\frac{x-X_{1}}{r_{1}} & \frac{y-Y_{3}}{r_{3}}-\frac{y-Y_{1}}{r_{1}}\\ \vdots & \vdots \\ \frac{x-X_{m}}{r_{m}}-\frac{x-X_{n}}{r_{n}} & \frac{y-Y_{m}}{r_{m}}-\frac{y-Y_{n}}{r_{n}}\\ \vdots & \vdots \end{array}\right]$$

Additional variable

(10) $$r_{m}=\sqrt{{\left(x-X_{m}\right)}^{2}+{\left(y-Y_{m}\right)}^{2}}$$

is the distance from the mobile terminal in point x to the mth base station. The iterative algorithm for finding a linear least squares estimate of mobile terminal coordinates can be summarized in equation [40]:

(11) $${\hat{x}}_{i+1}={\hat{x}}_{i}+{\left(A^{T}\left({\hat{x}}_{i}\right)G^{-1}A\left({\hat{x}}_{i}\right)\right)}^{-1}A^{T}\left({\hat{x}}_{i}\right)G^{-1}\left(r-d\left({\hat{x}}_{i}\right)\right)$$

where i is iteration number and iterations start from an initial guess ${\hat{x}}_{0}$.

#### 3.3.2. Levenberg–Marquardt

A reduction in the possibility of divergence in iterative position calculation can be achieved by using a method proposed by Levenberg [41] and Marquardt. It is based on a damped Gauss–Newton algorithm, which can be presented as an iterative equation:

(12) $${\hat{x}}_{i+1}={\hat{x}}_{i}+{\left(A^{T}\left({\hat{x}}_{i}\right)G^{-1}A\left({\hat{x}}_{i}\right)+\lambda_{i}I\right)}^{-1}A^{T}\left({\hat{x}}_{i}\right)G^{-1}\left(r-d\left({\hat{x}}_{i}\right)\right)$$

where I is the identity matrix. The $\lambda_{i}$ variable, called the damping parameter [42], controls the step size and reduces the risk of algorithm divergence. In our investigation, $\lambda_{i}$ was calculated in every step of the iteration using a simple algorithm presented in [39] in the form of a pseudo-code.

### 3.4. Neural Network Structure

**Figure 1:**
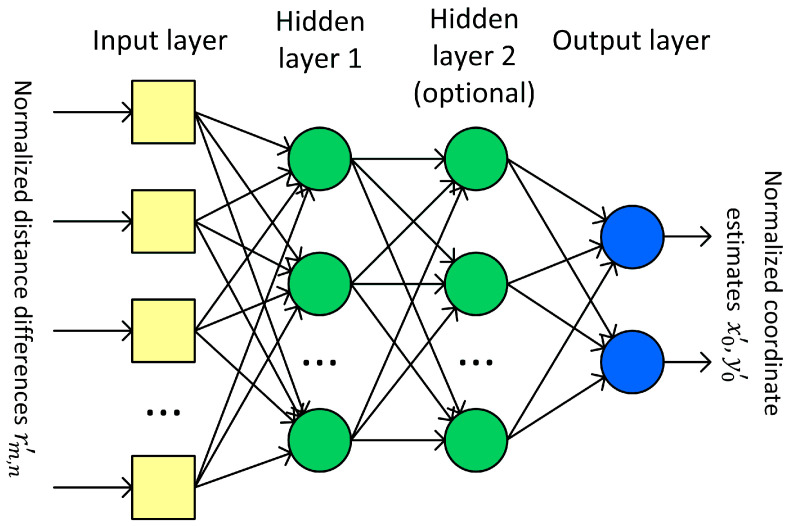
General structure of feedforward neural network used for initial coordinate estimation.

The feedforward neural network, also called the multilayer perceptron (MLP), was selected to solve the problem of the raw coordinates estimation for the first iteration in the iterative position calculation. MLP is able to model the functional relation between input signals and network output without knowing the function or its parameters [43]. The general structure of the neural network used in investigation is presented in Figure 1.

The input layer is linear and does not alter signals in any way—it only acts as a source of data for the first hidden layer. The size of the input layer equals the number of distance-difference values used to train the network. For N base stations, N−1 independent distance differences can be defined, but all possible combinations of $\left(N-1\right)(N-2)$ values were used as network input data because it allowed us to achieve faster network training without any impact on network performance after training. In the case of 3D positioning using cascaded networks, the second network (Section 4.2.3) uses the outputs of the first network in the cascade as input data, so the size of the input layer in the second network is increased by two.

Hidden layers are fully connected and they have non-linear activation functions. Various functions were tested; some of the best choices will be presented in Section 4. The output layer is linear or non-linear; the size of this layer equals the size of output data vector. In 2D positioning, it is always equal to two. However, in the case of a 3D position estimation, the networks used in different scenarios (Section 4.2) had output layer sizes equal to one, two or three.

All input and output data for neural networks were scaled in software to range from −1 to 1, as it is the default range of values for the most frequently used activation functions. The distance-difference values were normalized to the distance between corresponding base stations:

(13) $$r_{m,n}^{\prime }=\frac{\sqrt{{\left(x-X_{m}\right)}^{2}+{\left(y-Y_{m}\right)}^{2}}-\sqrt{{\left(x-X_{n}\right)}^{2}+{\left(y-Y_{n}\right)}^{2}}}{\sqrt{{\left(X_{m}-X_{n}\right)}^{2}+{\left(Y_{m}-Y_{n}\right)}^{2}}}$$

Also, the output from the network $x_{0}^{\prime },\;y_{0}^{\prime }$, which is an approximation of true coordinates x and y for the first iteration in iterative position estimation, was normalized to the size of the system operation area (400 m). Both normalizations should speed up the network training process; however, data normalization is optional, as a properly trained network should also be able to normalize data internally.

### 3.5. Network Efficiency Evaluation

The aim of the research carried out was to find the simplest possible structure of a feedforward neural network that allows us to estimate a coarse approximation of the position from TDoA measurements that can be used as a starting point for iterative position estimation algorithms. Iterative algorithms, supported by the proper selection of starting points, must achieve convergence to a correct result at each test point in the defined operating area of the system. The test point selection for all scenarios will be explained in Section 4. The reference scenario against which the results of using the neural network were compared was always the case where the coordinates of the origin of the coordinate system (x=0, y=0, z=0) were taken for the first iteration. Although the proper selection of a starting point allows us to reduce the number of iterations that are required to achieve a sufficiently small value of a position estimation update, typically used as stop condition, the number of iterations was recorded and presented, but was not taken into account as a parameter for neural network optimization. It may be a next step in network development in future works.

It should be stressed that a given neural network configuration was treated as sufficient when at least in one repetition of network training, repeated at least 10 times, the trained network gave a 100% convergence of position estimation algorithms. However, as the network training process is based on partially random operations (e.g., partially random generation of initial biases and weights for all neurons), the results of network training, even using the same training dataset, may differ in next repetitions of the training process. It frequently happened that the same neural network configuration resulted in 100% convergence efficiency in some simulations in the series, but in other simulations in several dozens of test points, the position estimation algorithms were divergent just because of small differences in weights and biases in FNN resulting from these partially random operations during training.

## 4. Simulations

### 4.1. 2D Case

**Figure 2:**
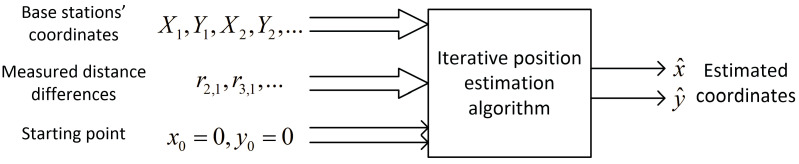
Data flow in the case of 2D iterative position estimation with fixed starting point.

**Figure 3:**
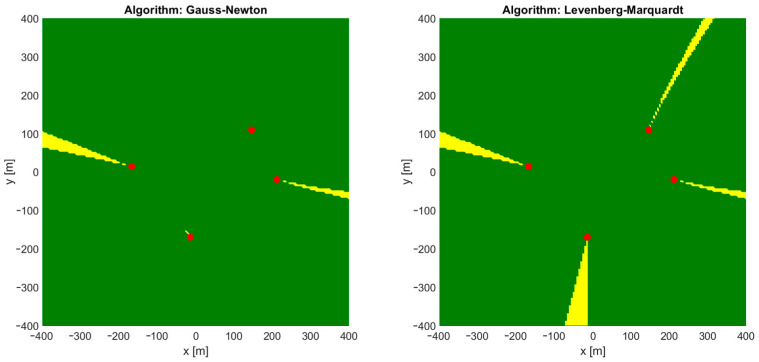
Regions of convergence for Gauss–Newton and Levenberg–Marquardt algorithms in 2D TDoA positioning. Green: correct convergence, yellow: convergence to incorrect position or divergence. Red dots: position of base stations.

In the simplest, two-dimensional scenario, differences in the height of base stations’ antennas and mobile nodes are omitted. Distance-difference values were calculated on an X–Y plane. Both the Gauss–Newton and the Levenberg–Marquardt iterative algorithms failed to provide correct position estimation results when the starting point for the first iteration was located in the origin of the local coordinate system, which means $x_{0}=0$, $y_{0}=0$. In such cases, a set of input data for a position estimation algorithm consists of base stations’ coordinates $X_{1},Y_{1},X_{2},Y_{2},\ldots$ and the results of distance-difference measurements $r_{2,1},r_{3,1},\ldots$ as presented in the block diagram in Figure 2. Regions in which these iterative algorithms returned incorrect coordinates or were even divergent are marked yellow on charts in Figure 3. It should be noted that some impact on the convergence may be seen when a different number of distance-difference equations are used in iterative algorithms. In the case of N base stations, only N−1 distance-difference values (4) are independent, while others are simply linear combinations that do not provide additional data. In the simulator used in our investigation, a full set of $\left(N-1\right)(N-2)$ positioning equations was used, and therefore all the results presented later in the paper were obtained for an overdetermined system of equations. However, it has been checked that conclusions on the usability of FNN to indicate initial coordinates for iterative position estimation are also valid when the numbers of position equations are reduced.

The results of a convergence check for the 2D TDoA positioning system are summarized in Table 3. The average and maximum number of iterations were counted only for cases which converged to the correct coordinates of the mobile terminal.

**Figure 4:**
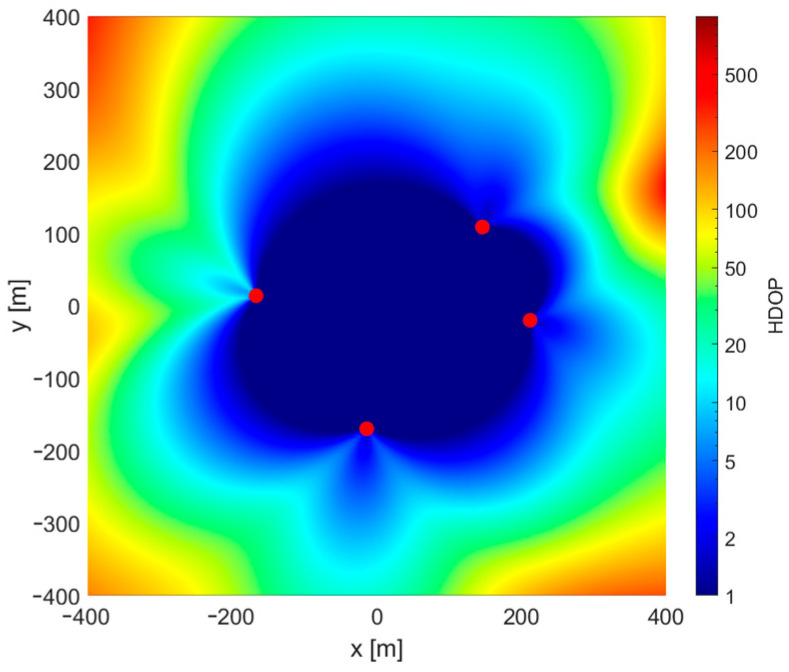
Dilution of precision chart for 2D TDoA scenario.

It is somehow surprising that there is no obvious coincidence of regions of incorrect convergence and regions with high values of a horizontal dilution of precision (HDOP), which is a good measure of relation between system geometry and possible position estimation accuracy. Comparing Figure 3 and Figure 4, it may be said that convergence problems occur mostly in regions located on the opposite side of one of the base stations from the starting point.

**Figure 5:**
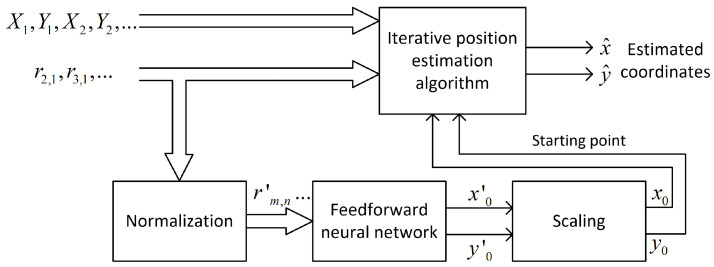
Data flow in the case of 2D iterative position estimation with starting point selection using feedforward neural network.

A feedforward neural network uses normalized distance-difference values (13) to estimate the scaled initial position of a mobile terminal $x_{0}^{\prime },y_{0}^{\prime }$ as it is presented in the block diagram in Figure 5. It was assumed that the learned network would only be used in one scenario, i.e., with an unchanging distribution of base stations. For this reason, the coordinates of the reference stations are not an input to the neural network.

To carry out the neural network learning process, it is necessary to prepare reference data. If the neural network is used as the sole tool to estimate position of a mobile object, the training dataset typically consists of reference data from points deployed uniformly over whole area of system operation and the goal of learning is to achieve the best quality of position estimation in terms of RMS or maximal position estimation error. But in our investigation, rough estimates of mobile terminal positions are used as starting points for fine position estimation using iterative algorithms. Therefore, although selecting the closest coordinates should usually result in a fast convergence with a low number of required iterations, correct convergence can be obtained also for an unlimited number of other possible candidates for starting points, even located far away from true terminal position. So, for the specified set of distance-difference data (1), there is more than one possible set of output data that allows us to achieve a convergence of iterative position estimation algorithms. It is also important that the closest approximation of the mobile terminal position may not be the best method of evaluation of the neural network effectiveness, because in the search for the simplest network structure, networks giving higher errors of position estimation may turn out to be better candidates. However, the definition of network training goals and the cost function for our investigation is not trivial, and finding the simplest FNN structure may require adaptive changes in training datasets during the training process, which is not already implemented in the tool used for FNN experiments. Therefore, our search for FNN structure was conducted by training the FNN using datasets with the true coordinates of the mobile terminal, selected uniformly and non-uniformly in a predefined area of system operation. It may of course raise question of whether the obtained results are really the simplest structures of FNN capable of solving the convergence problem, but, as it will be shown later, in all tested scenarios, the obtained network structures are really promising.

**Figure 6:**
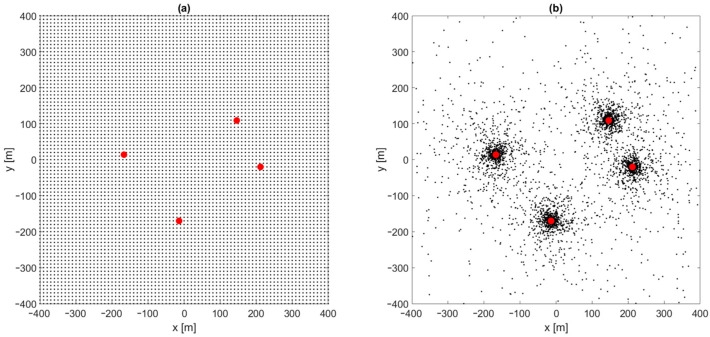
Uniform (**a**) and non-uniform (**b**) distribution of reference points for network training. Red dots: coordinates of base stations in TDoA positioning system.

The investigation of position estimation convergence improvement by FNN was started with a learning dataset consisting of reference distance-difference values for mobile terminal positions generated uniformly in the whole area of system operation in a grid with x and y coordinate step equal 10 m (Figure 6a). Therefore, for an area of 800 × 800 m, the reference dataset size was 6561. All simulations were made using Matlab (version R2022b) with a Deep Learning toolbox (version 14.5), using built-in network training procedures. Unless otherwise stated, the Levenberg–Marquardt algorithm with an RMS error function was used to train the network, with a random division of the reference dataset into three groups: 70% training data, 15% validation data and 15% test data. Please note that the Levenberg–Marquardt algorithm occurs twice in this article: as an iterative position estimation algorithm and as an FNN training algorithm. The initialization of neuron weights and biases in FNN was made using the Nguyen-Widrow algorithm, which contains some degree of randomness; therefore, repetitions of network training may result in different network parameters and performances. Thus, the presented data corresponds to the best results obtained in 10 to 30 repetitions of FNN training process. The performed simulations can be summarized in the following steps:

1. Generate test points uniformly distributed in the whole system area with an x and y step equal to 5 m (25,921 point in total), and calculate TDoA data for all test points;
2. Run the iterative position estimation algorithm (Gauss–Newton or Levenberg–Marquardt) with TDoA data corresponding to all test points, with the starting point in origin ($x_{0}=0$, $y_{0}=0$), and check convergence to correct coordinates;
3. Generate reference points for the neural network training: uniformly with an x and y step equal to 10 m (6561 points in total), or non-uniformly using the rules described later in the article; calculate TDoA data for all reference points;
4. Normalize TDoA data and reference points’ coordinates and train the feedforward neural network to predict normalized coordinates using normalized TDoA input data;
5. Verify the convergence of the iterative position estimation algorithm (G–N or L–M) using a larger set of test points from step 1 with initial coordinates calculated using the output of a neural network trained using a smaller set of training points from step 3.

Training the network using a smaller set of training points compared to the set of test points used to verify the convergence of iterative position estimation algorithms allows us to check its ability to generalize the solution by FNN. In this way, in step no. 5, the trained network is used to predict the coordinates of the mobile object using data that was not previously used to train the network.

Table 4 and Table 5 show the selected numerical results of the convergence analysis of the iterative algorithms with the starting points indicated by the neural networks, obtained using Gauss–Newton and Levenberg–Marquardt algorithms. The size of the input layer, which is 12, is determined by the number of distance-difference values $r_{m,n}$ for any m≠n. The input layer is always linear. The transfer function of the output layer is by default linear, but a non-linear case has also been checked. The size of the output layer equals the number of estimated coordinates. Various combinations of transfer functions have been checked in networks with one and two hidden layers. The symbol “lin” denotes the linear transfer function, “tanh” is the hyperbolic tangent function, “log” is the logistic sigmoid function, “ell” is the Elliot activation function and “rad” is the radial basis transfer function. For the selected neural network configuration: the number of layers, activation functions in all layers and for the two-layer case, the number of neurons in the second layer and the size of the first hidden layer was reduced by one starting from several dozen, until it was not possible to obtain a 100% convergence of position estimation. Then, another network configuration was selected (e.g., a different activation function) and the simulation process was repeated.

Data in both Table 4 and Table 5 are arranged in some subcategories that differ, e.g., by the number of hidden layers or activation functions in the first, the second or both hidden layers. Data in the rows printed in bold indicates the smallest network size (optimal configuration in terms of computational complexity) that achieved a 100% convergence of position estimation in these subcategories. In addition, some examples of neural networks much larger than required are also presented (e.g., the first three rows in Table 4) to show that an increasing accuracy of position estimation using FNN results in a slightly lower average number of iterations using both iterative algorithms (G–N and L–M). In turn, some examples of networks slightly smaller than the minimum network configurations marked in bold are also included to show that the degradation in the convergence ratio caused by reducing the FNN size below a critical value is not rapid and only causes convergence problems in a few test points out of over twenty-five thousand.

From the results presented in Table 4, even for networks with linear inputs and output layers and only one hidden layer with a hyperbolic tangent activation function, only eight neurons in hidden layers were necessary to obtain the convergence of the Gauss–Newton position estimation algorithm. A higher number of neurons in hidden layers obviously increased the quality of rough position estimation for the first iteration, but it had only a marginal impact on the average number of iterations needed to reach a final position estimation. On the other side, networks with seven or less neurons in hidden layers were not able to provide correct starting point coordinates in at least one of the tested points. It must be pointed out that a required hidden layer size of only eight neurons is already surprisingly small; however, it cannot be precluded that networks with even lower numbers of neurons but trained in different ways or with different structures are also able to estimate correct initial coordinates, reaching convergence in all test points. Thus, more tests were needed.

A change in the activation function in the output layer from a linear to hyperbolic tangent worsened the results, probably because the region of high variability of the tanh function values in output layers corresponds to the center of the area of the positioning system operation. Therefore, the output layer activation function was kept linear. The logistic sigmoid function in hidden layers gave almost exactly the same results as the hyperbolic tangent, which is not surprising as the logistic sigmoid function can be obtained by the proper scaling of tanh. From other activation functions that are differentiable in the entire domain, the Elliot sigmoid function and the radial basis function have been checked as potential candidates for hidden layers, but obtained results were significantly worse compared to hyperbolic tangent. Also, different network training algorithms and different error functions (mean error, sum of squared errors) could not outperform the results obtained using the Levenberg–Marquardt training algorithm with mean squared error as the error function. 

Further, tests were conducted with networks consisting of two hidden layers, both using a hyperbolic tangent activation function. The smallest configurations of hidden layers in terms of the total number of neurons with non-linear processing, which allows for obtaining a convergence of position estimations in all tested points, included the following: two layers with four neurons, a first layer with six neurons, followed by a second hidden layer with three neurons and first layer with twelve neurons, followed by a second layer with two neurons only. Any of these configurations could not outperform the network with one hidden layer working with eight neurons, so the minimal network configuration seems to be eight non-linear neurons, no matter if they are in one or two hidden layers.

The results of the convergence test for the Levenberg–Marquardt iterative position estimation algorithm, presented in Table 5, are a little better than those obtained using the Gauss–Newton algorithm in the case of FNN with two hidden layers, which is unexpected as the L–M algorithm with fixed starting points had a lower probability of convergence than G–N (see Table 3). For example, for the G–N algorithm, a minimal FNN configuration with four tanh neurons in the second layer required also four tanh neurons in the first hidden layer (a total of eight non-linear neurons, the smallest configuration of a two-layer network in this scenario) and three neurons in the second layer required six neurons in the first layer (nine in total). But, in the case of the L–M algorithm, four tanh neurons in the first hidden layer required only three neurons in the second layer (a total of seven non-linear neurons, the smallest configuration for the L–M algorithm) and the same number of neurons also worked correctly in a network with five neurons in the first layer and two in the second one (also seven in total). Therefore, in the case of the L–M algorithm, slightly smaller two-layer neural network configurations were found compared to the G–N case. However, when only one hidden layer was used, G–N slightly outperformed L–M in terms of convergence. Thus, no significant differences between G–N and L–M algorithms are visible.

**Figure 7:**
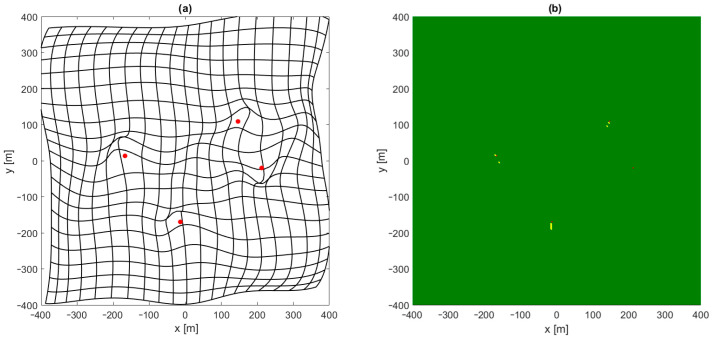
Example of deformation of coordinate grid (**a**) and results of iterative position estimation test (**b**) for Gauss–Newton algorithm. FNN trained with uniformly distributed data.

Interesting conclusions can be drawn by analyzing the distribution of places in which the convergence of position estimation algorithms was not achieved, even though a starting point selection by the neural network was used. In Figure 7a a kind of map is shown which presents the distortion of a mobile terminal coordinate grid estimated using a feedforward network. In the case of a perfect estimation, this figure should contain a grid of perpendicular straight lines with a 40 m raster. The deformation and displacement of lines clearly shows that the quality of position estimation using FNN is not the same in whole area of system operation. Comparing this map with Figure 7b, which presents the results of the convergence test, reveals that a lack of convergence is observed only in proximity to one or more base stations, and it coincides with some deformation in the grid in Figure 7a in these regions. Therefore, greater emphasis must be placed on the quality of position estimation using FNN close to all base stations. It may be achieved by changing the error function used during network training in such a way that in regions close to base stations, a lower position estimation error is required, but the simplest method would be to change the distribution of points used to generate reference data for network training while keeping the MSE error function unchanged.

The uneven deployment of reference points with a higher density close to base stations should increase the position estimation in these regions; therefore, the following procedure was proposed to generate a training dataset. The coordinates of candidate points were generated randomly with a uniform distribution in the whole area of system operation. Next, the distances $r_{n}$ from this candidate to all N base stations were calculated. The candidate is added to training dataset with a probability defined by equation:

(14) $$p=\max\left(1,{\max\left(\frac{1}{r_{n}^{2}}\right)}_{n=1\ldots N}\right)$$

Candidate generation is repeated until the predefined size of the training dataset is achieved. In order to obtain results comparable with those for a uniform distribution of training points, a dataset size of 6000 points was chosen. One of the obtained non-uniform distribution training points is presented in Figure 6b.

**Figure 8:**
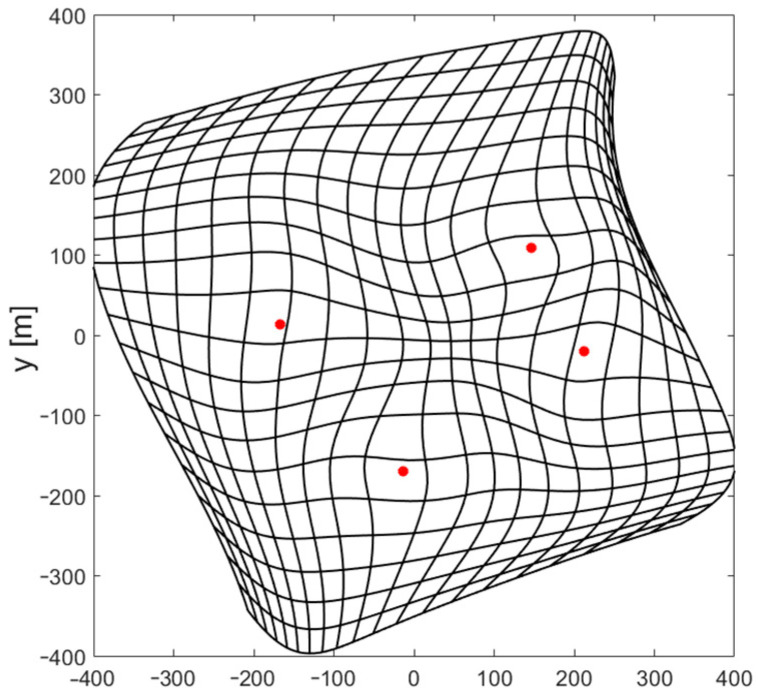
Example of deformation of coordinate grid for Gauss–Newton algorithm and FNN with four neurons in hidden layer. FNN trained with non-uniformly distributed data.

The results of using FNN with a non-uniform distribution of reference data used for training are very promising. Both the Gauss–Newton and Levenberg–Marquardt iterative position estimation algorithms were convergent in all tested points when the starting point for the first iteration was estimated using a neural network with only four neurons with a hyperbolic tangent activation function in the only hidden layer. Although, the numerical values of the position estimation for such a network were quite high, with an RMS error of 53.5 m and a maximum error exceeding 200 m, and though the deformation of the coordinate grid shown in Figure 8 is very large, especially near edges of the area of system operation, such a rough estimation of the position was sufficient to achieve the full convergence of both tested algorithms.

It is hard to imagine the possibility of solving the problem of estimating two coordinates of a mobile terminal with network consisting of less than four neurons with a non-linear activation function; therefore, the obtained network structure may be treated as the minimal FNN configuration. By extracting weights and biases from a trained network and reducing the number of input data to a minimal set of independent distance-difference values, it is possible to write equations that fully describe the operation of the minimal network structure found during the investigation:

(15) $$\begin{array}{ll} i & =\tanh\left(2.3735\cdot r_{2,1}-3.867\cdot r_{3,1}-0.0139\cdot r_{4,1}+3.7593\right)\\ j & =\tanh\left(3.0158\cdot r_{2,1}-2.0051\cdot r_{3,1}-0.7724\cdot r_{4,1}-3.1828\right)\\ k & =\tanh\left(2.2489\cdot r_{2,1}-0.0023\cdot r_{3,1}-3.26\cdot r_{4,1}+3.0828\right)\\ l & =\tanh\left(-0.3013\cdot r_{2,1}-1.1367\cdot r_{3,1}-1.3176\cdot r_{4,1}-2.966\right) \end{array}$$

(16) $$\begin{array}{c} x_{0}^{\prime }=-8.8602\cdot i+4.9286\cdot j+3.4307\cdot k-4.0567\cdot l+6.4201\\ y_{0}^{\prime }=2.7504\cdot i+9.9756\cdot j-1.0157\cdot k-9.8887\cdot l-1.6524 \end{array}$$

Comparing the data from Table 3 and Table 6, it is also obvious that the pre-calculation of starting points significantly reduces the average and maximal number of iterations needed to reach the assumed stop condition in iterative position calculation, typically defined as the maximal norm of the position update vector, which was 10^−4^. The gain in the reduced number of iterations fully compensates the additional computational effort that is caused by the need to calculate starting points using (15) and (16).

### 4.2. 3D Case

In 3D hyperbolic positioning, at least five reference points are needed to obtain an unambiguous position indication. However, when these reference points have coordinates of stations no. 1 to 5 from Table 2, the maximum value of 3D dilution of precision parameter (DOP) exceeds 2.5 million in the area to the north of station no. 5 and to the south of station no. 4. A positioning system with such a high value of DOP is practically unusable, as a 1 mm error in distance-difference measurements could cause a 2.5 km position estimation error. It has been checked that even with such an unfavorable geometry of base stations, it is possible to train FNN to indicate starting points for the G–N algorithm using, e.g., two separate networks from Section 4.2.2; the first network: one layer tanh, 40 neurons and the second one: one layer tanh, 400 neurons. However, in order to make the results more usable, another base station has been added and all the 3D simulation results presented in next subsections were obtained using six base stations. In such a case, the maximum DOP was 845, which is still not low enough to reach a high position estimation quality in whole area of system operation, but can be accepted in some regions in practical solutions.

**Figure 9:**
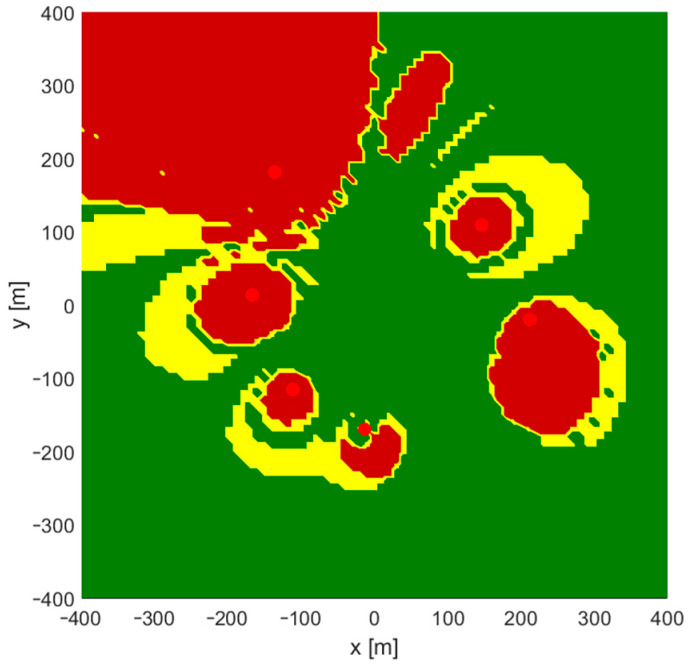
Regions of convergence for Gauss–Newton algorithms in 3D TDoA positioning. Green: correct convergence, yellow: convergence to incorrect position or divergence at least for one value of *z* coordinate, dark red: lack of convergence for any value of *z*. Red dots: position of base stations.

Test points for the 3D scenario were distributed uniformly with x  and y in a range from −400 to 400 m and an step equal to 10 m. The z range was set from 0 to 20 m with a step equal to 2 m. After removing points located too close to base stations, a total number of 72,164 points were checked for the convergence of the Gauss-Newton iterative position calculation algorithm. The results obtained for the fixed starting point $x_{0}=0,\;y_{0}=0,\;z_{0}=0$ are depicted in Figure 9, where a yellow color indicates that at least for one value of z coordinate the G–N algorithm was divergent or converged to an incorrect result, while the dark red color indicates regions where convergence was not possible for any value of z. In this scenario, convergence was achieved in 49,365 points, which is 68.4% of test points.

The results presented in Section 4.1 for 2D positioning showed that the G–N algorithm supported by a feedforward neural network with only one hidden layer and hyperbolic tangent activation function allowed us to obtain representative results, compared to other network structures. Thus, in this section, only the results obtained for the G–N algorithm and one-layer networks with a tanh function will be presented, although different network structures and functions were also tested. It should be noted that the hyperbolic tangent activation function is also frequently used by other authors, e.g., in direction of arrival estimation presented in [28].

#### 4.2.1. One Network

Compared to the 2D case presented in Figure 5, the indication of starting points in 3D positioning system requires only adding one more neuron in the output layer of the feedforward neural network. However, in this straightforward approach, there is no way to control what part of a neural network resource is used to estimate horizontal and vertical coordinates.

**Figure 10:**
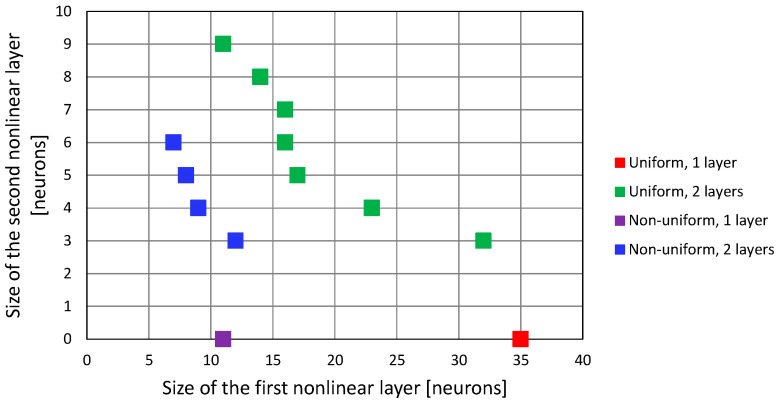
Configuration of FNN that results in convergence of 3D G–N algorithm in all test points.

The minimal configurations of neural networks with one and two hidden layers with a hyperbolic tangent activation function, that allowed us to achieve convergence in all test points in the 3D case, are presented in Figure 10 for both the linear and non-linear deployment of reference points in the network training dataset. Assuming that the computation of a non-linear activation function value is the most resource-consuming part of FNN work, the closer the points are to the origin in Figure 10, the less complicated the network structure is.

When the feedforward neural network was trained using a uniform distribution of the training dataset, networks with only one hidden layer required 35 tanh neurons, while two-layered networks required 11 neurons in the first hidden layer and 9 in the second one, amounting to 20 non-linear neurons in total. However, the non-uniform distribution of training data allowed us to obtain the same result (convergence of G–N algorithm in all test points) using 11 tanh neurons in only one hidden layer. Adding the second layer did not improve the results, as the minimum configurations of a two-layer FNN trained using a non-uniform dataset required 13 tanh neurons in total (configuration: 7 + 6, 8 + 5 and 9 + 4 neurons).

#### 4.2.2. Two Separate Networks

**Figure 11:**
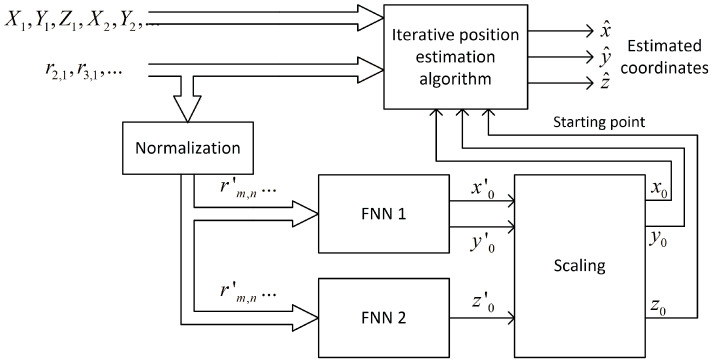
Data flow in case of 3D iterative position estimation with starting point selection using two separate feedforward neural networks.

The range of x and y coordinates in the scenario presented in Section 4.2 differs significantly from range of z. Thus, the required accuracy of the initial values of $x_{0},\;y_{0}$ and $z_{0}$ may also be different. Two separate neural networks were implemented to check if a separate estimation of horizontal and vertical coordinates allows us to reduce the computational complexity understood as the total number of non-linear neurons. The block diagram of position estimation in this experiment is depicted in Figure 11.

**Figure 12:**
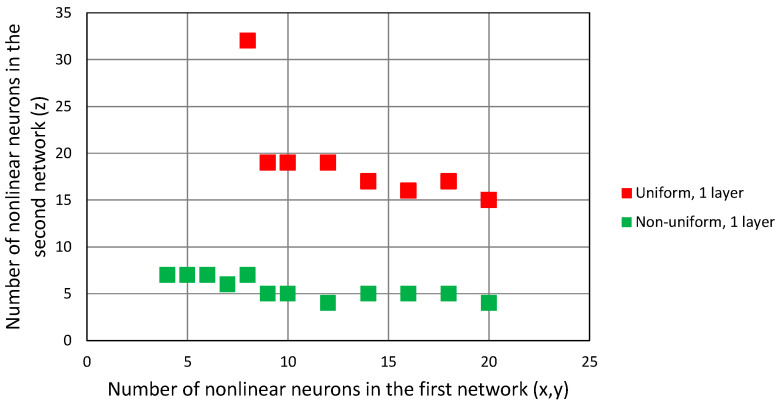
Configuration of two independent neural networks that result in convergence of 3D G–N algorithm in all test points.

The results of the simulations are presented in Figure 12 in form of points indicating the number of non-linear neurons in the first network, responsible for the horizontal coordinate estimation and the number of non-linear neurons in the second network, which was only for estimating vertical coordinates. Both networks had only one hidden layer.

In the case of the uniform distribution of data in training dataset, the minimal size of both FNN, in terms of the total number of non-linear neurons, was 28, but the majority of them (19) had to be in the second network, responsible for z coordinate estimation. This clearly indicates a greater importance of this coordinate in the convergence of the G–N algorithm. The same trend is visible in the data from the networks trained using a non-uniform dataset: the minimal configuration of networks was four neurons in the *x*-*y* network and seven neurons in z network, amounting to 11 in total. However, comparing these results to the case with one network only, no reduction in the number of neurons in the smallest network configuration was observed.

#### 4.2.3. Two Networks Cascaded

**Figure 13:**
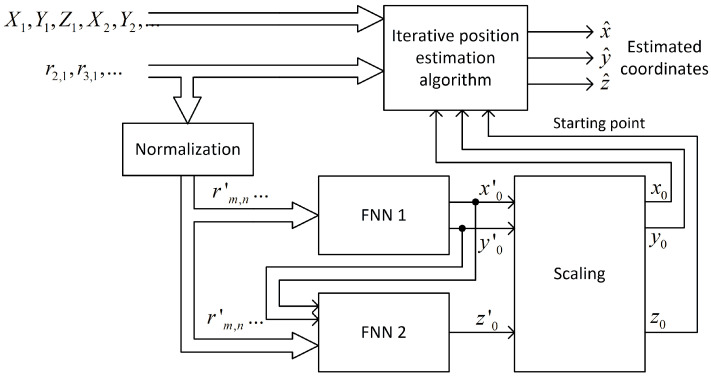
Data flow in the case of 3D iterative position estimation with starting point selection using two cascaded feedforward neural networks.

Another neural network configuration that has been tested in simulations is presented in Figure 13. Also, this time, two feedforward neural networks have been used, but the second network, responsible for vertical coordinate estimation, was able to use output data from the first network as additional inputs, so these networks are connected in a cascade.

**Figure 14:**
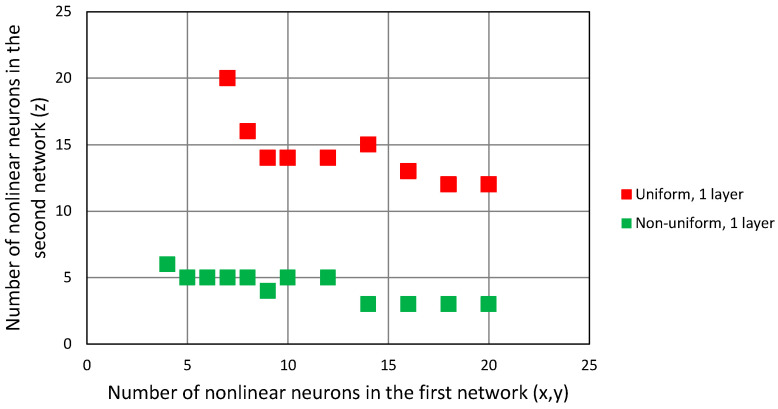
Configuration of two cascaded neural networks that result in convergence of 3D G–N algorithm in all test points.

In the cascaded configuration, the second FNN takes advantage of the output from the first network, and therefore is able to provide a comparable quality of prediction using the lower number of non-linear neurons. Comparing the results from Figure 14 with Figure 12, it can be seen that almost in all cases for the same size of the first network, the number of non-linear neurons in the second FNN required for the convergence of G–N algorithm is reduced. The minimal configuration of two cascaded networks in the case of the uniform training dataset is 9 + 14 neurons, and in the case of the non-uniform distribution of training points, it is 5 + 5 neurons. It should be noted that it still indicates a higher computational complexity of vertical coordinate estimation, as the first FNN with five neurons in the hidden layer estimates two coordinates, while the second FNN needs five neurons for one coordinate.

## 5. Conclusions

When neural networks are used for position estimation instead of dedicated algorithms, iterative or closed-form, usually a larger network results in a better accuracy of the obtained results. Therefore, it is not surprising that, for example, in [44], a multi-layer perceptron with up to 1200 neurons was used to estimate the position in a MIMO system based on channel state information. But, when a neural network is only used for coarse position estimation, used as a starting point for fine position calculation using iterative algorithms, much smaller networks with only a dozen of non-linear neurons turned out to be sufficient. However, compared to the results of other investigations presented in the literature, such small network configurations are not surprising. For example, in [28], in a solution based on direction of arrival (DoA) estimation, the neural network consisted of two layers with a dozen neurons each, while in [20], FNN with 10 neurons in the first layer and 20 in the second one was successfully used for RSS-based position estimation. Even smaller neural networks with no more than five neurons were tested in [18] in a small positioning network based on RSS measurements. Therefore, the FNN structures presented in this article are consistent with the literature.

One of the most important conclusions from the investigation presented in Section 4 is that when the accuracy of position estimation in different parts of system’s area of operation is not the same, proper preparation of the dataset for training the network can significantly reduce the size of the feedforward neural network that is necessary to achieve the objective, which in our case was the convergence of the position estimation algorithm. The non-uniform distribution of points in a training dataset, with a focus on the critical area for convergence, allowed us to reduce the FNN size to less than half of a network trained using uniformly distributed points, both in 2D and 3D positioning. However, it should be emphasized that all the neural networks used in our simulations were trained for the base stations’ configurations shown in Table 2 and will not work for other reference stations’ deployment without retraining. But, the tacit assumption that the case-study solution is acceptable can be found in most publications on neural networks and deep learning in position estimation, including the articles mentioned in Section 2. Generalizing a solution that can estimate a position in any possible configuration of reference stations will certainly require much larger neural networks and it may be interesting topic for future work.

Another topic worth investigating in the future is the rate of the convergence of iterative position estimation algorithms. In this paper, the number of iterations was not taken into account in the network training process because the error function was the mean squared error of coordinates estimated by the neural network with respect to the actual coordinates of the test point. However, when the size of the FNN is already defined, the network training process can be evaluated by the speed of convergence to reduce the total computational complexity of both the neural network and the iterative position calculation algorithm.

## Figures and Tables

**Table 1:** A summary of neural network-based position estimation solutions.

Work	Year	Environment	Radio Interface	Meas. Param.	Network Type and Size	Netw. Use	Performance	Comments
Mok et al. [12]	2013	University building	Wi-Fi	RSS (M)	FNN/MLP, 1 L/5 N	PE	MSE 2.6 to 23 m	
Narita et al. [13]	2021	7 × 4 m room	Wi-Fi	RSS (M)	FNN/MLP, 5 L/500 N	PE/FP	Avg. 0.93 m, max. 6.7 m	
Paudel et al. [14]	2022	2 rooms in univ. building	Wi-Fi dual band	RSS (M)	SVR, LR, KNN, other	PE/FP	Avg. 2.2 m	
Urwan et al. [15]	2022	Indoor (university building) and outdoor	Wi-Fi and LTE	RSS (M)	FNN/MLP 2–5 L/169–311 N	PE/FP	0.9 to 25 m indoor, 12.7 to 55.4 m outdoor	Separate models for indoor and outdoor
Bhatti [16]	2018	100 × 100 m	Simulation	RSS (S)	LR/SVM	PE	Approx. 0.6 m	
Alhmiedat [17]	2023	University lab, 21 × 7.6 m	ZigBee	RSS (M)	LR/KNN/DT/RF	PE	ME 1.4–4.6 m	
Gadhgadhi et al. [18]	2020	10 × 10 m	No data	RSS (S)	FNN/MLP 1 L/3–4 N	PE	1.1 m	
Al-Tahmeesschi et al. [19]	2022	Outdoor, Madrid simulator	5G mmWave	RSS (S)	KNN/MLP/LSTM 3 L/1024/512/64 N	PE	ME: 0.5–5.4	
Avellaneda et al. [20]	2023	Two-bedroom apartment	BLE	RSS (M)	FNN/MLP 2 L/10 + 20 N	PE/Class	88% to 97% class. prob.	
Zheng et al. [21]	2021	Laboratory 10 × 20.6 m	EnOcean	RSS (M)	FNN/SVM 1–5 L/4 N	Class	96% class. prob.	
Dvorecki et al. [26]	2019	Office 45 × 25 m	Wi-Fi	RTT (M)	FNN 6L/228/50/251 N	FE	Mean range error 0.7–2 m	
Guidara et al. [27]	2021	Laboratory 9 × 9 m	868 MHz	RSS, LQI, T, RH (M)	FNN/LP 1–5 L/4–8 N	FE	Mean range error 0.92 m	
Malmström et al. [28]	2019	Outdoor, urban area	5G 15 GHz	RSRP (M)	FNN/RF 2 L/12–16 N	PE	ME 2–39 m	Antenna array 8 × 8
Gong et al. [29]	2022	Outdoor 50 × 50 m	MIMO OFDM	CSI (S)	FNN/MLP 2 L/128 + 128 N	PE/FP	ME 0.4–0.9 m	Linear antenna array
Kotrotsios et al. [30]	2021	One apartment	BLE	RSS (M)	FNN/MLP 2 L/64 N	FE	ME 0.7 m	
Cho et al. [31]	2019	Outdoor 6 × 6 km	LoRa	TDoA (S)	FNN	FE	ME 61 m	TDoA data conditioning
Wu et al. [32]	2018	10 × 10 m	No data	TDoA (S)	SVM	FE	Up to 99% LOS/NLOS det. prob.	LOS/NLOS identification

Measured parameters: RSS—received signal strength, RTT—round-trip time, LQI—link quality indicator, T—temperature, RH—humidity, RSRP—received signal reference power, CSI—channel state information, TDoA—time difference of arrival, (M)—measurements, (S)—simulations. Network type: FNN—feedforward neural network, MLP—multilayer perceptron, SVR—support vector regression, LR—linear regression, KNN—K-nearest neighbor, SVM—support vector machine, DT—decision tree, RF—random forest, LSTM—long-short term memory, xL/xN—number of hidden layers/neurons. Network use: PE—position estimation, FP—fingerprinting, Class—classification, FE—feature extraction.

**Table 2:** Coordinates of base stations in the local Cartesian coordinate system.

Station Number	X [m]	Y [m]	Z [m]	2D	3D
1	146	109	20	✓	✓
2	−14	−170	27	✓	✓
3	−167	14	26	✓	✓
4	212	−20	23	✓	✓
5	−136	181	15		✓
6	−112	−116	20		✓

**Table 3:** Results of convergence check for 2D TDoA system with fixed starting point coordinates.

Position Estimation Algorithm	Test Point Step (x/y)	Total Number of Test Points	Points with Correct Convergence	Probability of Correct Convergence	Average Number of Iterations	Maximum Number of Iterations
Gauss-Newton	5 m	25,921	25,648	98.946%	8.007	92
Levenberg–Marquardt	5 m	25,921	25,189	97.176%	12.89	94

**Table 4:** Convergence of iterative position estimation using Gauss–Newton algorithm with starting points estimated using FNN trained using uniform dataset.

FNN						Iterative Position Estimation Convergence				
Input Layer	Hidden Layer 1	Hidden Layer 2	Output Layer	Position Estimation RMS Error [m]	Position Estimation Maximum Error [m]	Tested Points	Correct Convergence	Probability of Convergence	Average Number of Iterations	Maximum Number of Iterations
12: lin	50: tanh	-	2: lin	1.27	12.59	25,921	25,921	100	3.95	12
12: lin	30: tanh	-	2: lin	1.61	9.87	25,921	25,921	100	3.98	11
12: lin	15: tanh	-	2: lin	10.91	62.64	25,921	25,921	100	4.47	12
12: lin	10: tanh	-	2: lin	14.43	63.38	25,921	25,921	100	4.67	15
**12: lin**	**8: tanh**	**-**	**2: lin**	**18.31**	**66.6**	**25,921**	**25,921**	**100**	**4.81**	**16**
12: lin	7: tanh	-	2: lin	18.93	73.47	25,921	25,920	99.996	4.84	15
**12: lin**	**20: tanh**	**-**	**2: tanh**	**6.63**	**41.88**	**25,921**	**25,921**	**100**	**4.24**	**16**
12: lin	15: tanh	-	2: tanh	15.64	71.4	25,921	25,920	99.996	4.69	10
12: lin	10: log	-	2: lin	10.73	55.74	25,921	25,921	100	4.5	56
**12: lin**	**7: log**	**-**	**2: lin**	**24.25**	**93.25**	**25,921**	**25,921**	**100**	**4.94**	**8**
12: lin	6: log	-	2: lin	26.25	109.9	25,921	25,920	99.996	4.94	8
**12: lin**	**15: ell**	**-**	**2: lin**	**5.66**	**31.15**	**25,921**	**25,921**	**100**	**4.22**	**15**
12: lin	12: ell	-	2: lin	9	57	25,921	25,920	99.996	4.41	16
**12: lin**	**20: rad**	**-**	**2: lin**	**6.36**	**38.13**	**25,921**	**25,921**	**100**	**4.22**	**8**
12: lin	15: rad	-	2: lin	10.49	45.8	25,921	25,920	99.996	4.43	21
12: lin	10: tanh	4: tanh	2: lin	6.05	26.26	25,921	25,921	100	4.24	15
**12: lin**	**4: tanh**	**4: tanh**	**2: lin**	**16.13**	**76.97**	**25,921**	**25,921**	**100**	**4.73**	**19**
**12: lin**	**6: tanh**	**3: tanh**	**2: lin**	**11.61**	**55.87**	**25,921**	**25,921**	**100**	**4.5**	**10**
12: lin	5: tanh	3: tanh	2: lin	19.98	77.67	25,921	25,920	99.996	4.83	10
**12: lin**	**12: tanh**	**2: tanh**	**2: lin**	**9.15**	**41.42**	**25,921**	**25,921**	**100**	**4.42**	**11**
12: lin	10: tanh	2: tanh	2: lin	13.1	62.1	25,921	25,919	99.992	4.59	12

**Table 5:** Convergence of iterative position estimation using Levenberg–Marquardt algorithm with starting points estimated using FNN trained using uniform dataset.

FNN						Iterative Position Estimation Convergence				
Input Layer	Hidden Layer 1	Hidden Layer 2	Output Layer	Position Estimation RMS Error [m]	Position Estimation Maximum Error [m]	Tested Points	Correct Convergence	Probability of Convergence	Average Number of Iterations	Maximum Number of Iterations
12: lin	50: tanh	-	2: lin	0.93	7.46	25,921	25,921	100	10.17	13
12: lin	30: tanh	-	2: lin	1.63	9.51	25,921	25,921	100	10.33	14
12: lin	15: tanh	-	2: lin	6.94	33.03	25,921	25,921	100	10.67	14
**12: lin**	**10: tanh**	**-**	**2: lin**	**21.97**	**95.25**	**25,921**	**25,921**	**100**	**10.92**	**19**
12: lin	8: tanh	-	2: lin	18.73	77.25	25,921	25,920	99.996	10.86	16
12: lin	8: tanh	4: tanh	2: lin	10.14	47.67	25,921	25,921	100	10.75	19
12: lin	4: tanh	4: tanh	2: lin	22.88	85.7	25,921	25,921	100	10.94	16
**12: lin**	**4: tanh**	**3: tanh**	**2: lin**	**25.06**	**88.4**	**25,921**	**25,921**	**100**	**10.96**	**14**
**12: lin**	**5: tanh**	**2: tanh**	**2: lin**	**34.6**	**137.5**	**25,921**	**25,921**	**100**	**10.99**	**19**
12: lin	4: tanh	2: tanh	2: lin	34.95	119.3	25,921	25,917	99.98	10.96	18

**Table 6:** Results of convergence test in the case of starting point estimation by FNN with four neurons in hidden layer, trained using non-uniform distribution of training data.

Position Estimation Algorithm	Iterative Position Estimation Convergence				
	Tested Points	Correct Convergence	Probability of Convergence	Average Number of Iterations	Maximum Number of Iterations
Gauss–Newton	25,921	25,921	100	5.19	7
Levenberg–Marquardt	25,921	25,921	100	11.11	15

## Data Availability

Data are contained within the article.

## References

[1] Naseem A., Rehman M.A., Abdeljawad T. (2020). Numerical Algorithms for Finding Zeros of Nonlinear Equations and Their Dynamical Aspects. J. Math..

[2] Marquardt D.W. (1963). An Algorithm for Least-Squares Estimation of Nonlinear Parameters. J. Soc. Ind. Appl. Math..

[3] Chan Y.T., Ho K.C. (1994). A Simple and Efficient Estimator for Hyperbolic Location. IEEE Trans. Signal Process..

[4] Fang B.T. (1990). Simple Solutions for Hyperbolic and Related Position Fixes. IEEE Trans. Aerosp. Electron. Syst..

[5] Bucher R., Misra D. (2002). A Synthesizable VHDL Model of the Exact Solution for Three-dimensional Hyperbolic Positioning System. VLSI Des..

[6] Sirola N. Closed-form algorithms in mobile positioning: Myths and misconceptions. Proceedings of the 2010 7th Workshop on Positioning, Navigation and Communication.

[7] Czapiewska A., Sadowski J. Analysis of Accuracy of Modified Gradient Method in Indoor Radiolocalisation System. Proceedings of the 2014 IEEE 79th Vehicular Technology Conference (VTC Spring).

[8] Ghorpade S., Zennaro M., Chaudhari B. (2021). Survey of Localization for Internet of Things Nodes: Approaches, Challenges and Open Issues. Future Internet.

[9] Laoudias C., Moreira A., Kim S., Lee S., Wirola L., Fischione C. (2018). A Survey of Enabling Technologies for Network Localization, Tracking, and Navigation. IEEE Commun. Surv. Tutor..

[10] Khan H., Hayat M.N., Ur Rehman Z. Wireless sensor networks free-range base localization schemes: A comprehensive survey. Proceedings of the 2017 International Conference on Communication, Computing and Digital Systems (C-CODE).

[11] Dwivedi A., Vamsi P.R. Performance analysis of range free localization methods for wireless sensor networks. Proceedings of the 2017 4th International Conference on Signal Processing, Computing and Control (ISPCC).

[12] Mok E., Cheung B.K.S. (2013). An Improved Neural Network Training Algorithm for Wi-Fi Fingerprinting Positioning. ISPRS Int. J. Geo-Inf..

[13] Narita Y., Lu S., Kamabe H. Accuracy Evaluation of Indoor Positioning by Received Signal Strength using Deep Learning. Proceedings of the 2022 24th International Conference on Advanced Communication Technology (ICACT).

[14] Paudel K., Kadel R., Guruge D.B. (2022). Machine-Learning-Based Indoor Mobile Positioning Using Wireless Access Points with Dual SSIDs—An Experimental Study. J. Sens. Actuator Netw..

[15] Urwan S., Wysocka D.R., Pietrzak A., Cwalina K.K. (2022). Position Estimation in Mixed Indoor-Outdoor Environment Using Signals of Opportunity and Deep Learning Approach. Int. J. Electron. Telecommun..

[16] Bhatti G. (2018). Machine Learning Based Localization in Large-Scale Wireless Sensor Networks. Sensors.

[17] Alhmiedat T. (2023). Fingerprint-Based Localization Approach for WSN Using Machine Learning Models. Appl. Sci..

[18] Gadhgadhi A., HachaΪchi Y., Zairi H. A Machine Learning based Indoor Localization. Proceedings of the 2020 4th International Conference on Advanced Systems and Emergent Technologies (IC_ASET).

[19] Al-Tahmeesschi A., Talvitie J., López–Benítez M., Ruotsalainen L. Deep Learning-based Fingerprinting for Outdoor UE Positioning Utilising Spatially Correlated RSSs of 5G Networks. Proceedings of the 2022 International Conference on Localization and GNSS (ICL-GNSS).

[20] Avellaneda D., Mendez D., Fortino G. (2023). A TinyML Deep Learning Approach for Indoor Tracking of Assets. Sensors.

[21] Zheng B., Masuda T., Shibata T. An Indoor Positioning with a Neural Network Model of TensorFlow for Machine Learning. Proceedings of the 2021 International Symposium on Intelligent Signal Processing and Communication Systems (ISPACS).

[22] Bellavista-Parent V., Torres-Sospedra J., Pérez-Navarro A. (2022). Comprehensive Analysis of Applied Machine Learning in Indoor Positioning Based on Wi-Fi: An Extended Systematic Review. Sensors.

[23] Feng X., Nguyen K.A., Luo Z. (2022). A survey of deep learning approaches for WiFi-based indoor positioning. J. Inf. Telecommun..

[24] Li Z., Xu K., Wang H., Zhao Y., Wang X., Shen M. (2019). Machine-Learning-Based Positioning: A Survey and Future Directions. IEEE Netw..

[25] Alhomayani F., Mahoor M.H. (2020). Deep learning methods for fingerprint-based indoor positioning: A review. J. Locat. Based Serv..

[26] Dvorecki N., Bar-Shalom O., Banin L., Amizur Y. A Machine Learning Approach for Wi-Fi RTT Ranging. Proceedings of the International Technical Meeting of The Insitute of Navigation ION ITM 2019.

[27] Guidara A., Fersi G., Jemaa M.B., Derbel F. (2021). A new deep learning-based distance and position estimation model for range-based indoor localization systems. Ad Hoc Netw..

[28] Malmström M., Skog I., Razavi S.M., Zhao Y., Gunnarsson F. 5G Positioning—A Machine Learning Approach. Proceedings of the 2019 16th Workshop on Positioning, Navigation and Communications (WPNC).

[29] Gong X., Yu X., Liu X., Gao X. (2022). Machine Learning-Based Fingerprint Positioning for Massive MIMO Systems. IEEE Access.

[30] Kotrotsios K., Orphanoudakis T. Accurate Gridless Indoor Localization Based on Multiple Bluetooth Beacons and Machine Learning. Proceedings of the 2021 7th International Conference on Automation, Robotics and Applications (ICARA).

[31] Cho J., Hwang D., Kim K.-H. Improving TDoA Based Positioning Accuracy Using Machine Learning in a LoRaWan Environment. Proceedings of the 2019 International Conference on Information Networking (ICOIN).

[32] Wu C., Hou H., Wang W., Huang Q., Gao X. TDOA Based Indoor Positioning with NLOS Identification by Machine Learning. Proceedings of the 2018 10th International Conference on Wireless Communications and Signal Processing (WCSP).

[33] Nessa A., Adhikari B., Hussain F., Fernando X.N. (2020). A Survey of Machine Learning for Indoor Positioning. IEEE Access.

[34] Isaia C., Michaelides M.P. (2023). A Review of Wireless Positioning Techniques and Technologies: From Smart Sensors to 6G. Signals.

[35] Kabiri M., Cimarelli C., Bavle H., Sanchez-Lopez J.L., Voos H. (2023). A Review of Radio Frequency Based Localisation for Aerial and Ground Robots with 5G Future Perspectives. Sensors.

[36] Shen G., Zetik R., Thoma R.S. Performance comparison of TOA and TDOA based location estimation algorithms in LOS environment. Proceedings of the 2008 5th Workshop on Positioning, Navigation and Communication.

[37] Yan J., Tiberius C., Bellusci G., Janssen G. Feasibility of Gauss-Newton method for indoor positioning. Proceedings of the 2008 IEEE/ION Position, Location and Navigation Symposium.

[38] Bancroft S. (1985). An Algebraic Solution of the GPS Equations. IEEE Trans. Aerosp. Electron. Syst..

[39] Mensing C., Plass S. Positioning Algorithms for Cellular Networks Using TDOA. Proceedings of the 2006 IEEE International Conference on Acoustics Speech and Signal Processing Proceedings.

[40] Foy W.H. (1976). Position-Location Solutions by Taylor-Series Estimation. IEEE Trans. Aerosp. Electron. Syst..

[41] Levenberg K. (1944). A method for the solution of certain non-linear problems in least squares. Q. Appl. Math..

[42] Umar A.O., Sulaiman I.M., Mamat M., Waziri M.Y., Zamri N. (2020). On damping parameters of Levenberg-Marquardt algorithm for nonlinear least square problems. J. Phys. Conf. Ser..

[43] Goodfellow I., Bengio Y., Courville A. (2016). Deep Learning.

[44] Sobehy A. (2020). Machine Learning Based Localization in 5G. Ph.D. Thesis.

